# The optimal range of serum intact parathyroid hormone for a lower risk of mortality in the incident hemodialysis patients

**DOI:** 10.1080/0886022X.2021.1903927

**Published:** 2021-03-29

**Authors:** Xiaoling Zhou, Yidan Guo, Yang Luo

**Affiliations:** Department of Nephrology, Beijing Shijitan Hospital, Capital Medical University, Beijing, China

**Keywords:** Parathyroid hormone, mortality, dialysis

## Abstract

The serum intact parathyroid hormone (iPTH) is associated with the prognosis of hemodialysis (HD) patients, however, its optimal range for reducing mortality remains inconsistent. We designed a prospective cohort study of 346 incident HD patients to assess the association between different serum iPTH level and mortality. According to the Kidney Disease Outcomes Quality Initiative (K/DOQI) international guidelines (2003), we divided patients into three groups (iPTH < 150 pg/mL, 150–300 pg/mL and >300 pg/mL). During the median follow-up of 58 months, 157 patients (45.38%) died. Multivariate Cox regression analysis showed that iPTH < 150 pg/mL and >300 pg/mL were associated with all-cause and cardiovascular mortality. Then, we performed a sensitivity analysis of patients divided into 6 serum PTH levels groups according to the folds of the K/DOQI target range. Multivariate Cox regression analysis showed that patients with serum iPTH ≥750 pg/mL, 600–749 pg/mL, 450–599 pg/mL had significantly higher risk of all-cause and cardiovascular mortality compared with those with serum iPTH in the range of 150–299 pg/mL. The association between serum iPTH and mortality shows a *U*-shaped curve. The optimal serum iPTH level which confers the lowest risk of all-cause and cardiovascular mortality could range from 150 pg/mL to 450 pg/mL in this group of incident HD patients.

## Introduction

Despite therapeutic development, chronic kidney disease-mineral and bone disorder (CKD-MBD) is still a critical issue among patients undergoing hemodialysis (HD) [1–4]. Several large observational studies have indicated that the occurrence of CKD-MBD is associated with an increased risk of mortality [5–9]. In 2003, the US National Kidney Foundation implemented the Kidney Disease Outcomes Quality Initiative (K/DOQI) international guidelines to establish target levels for serum intact parathyroid hormone (iPTH), calcium (Ca), and phosphorus (P) to help lower the CKD-MBD-related mortality. However, evidence suggested that this restrictive guideline was difficult to achieve, especially over the long term of HD treatment [10–13].

After that guideline was recommended, the Kidney Disease Improving Global Outcomes (KDIGO) published a new CKD-MBD guideline in 2009. The guideline suggested maintaining serum iPTH level in the range of approximately two to nine times the upper normal limit for the assay (2 C) [14]. Since the guideline was implemented, there has been an increased awareness of CKD-MBD, however, the level of evidence for this iPTH level recommendation is only C, which indicates a lack about the impact of different levels of serum iPTH on mortality. Also, the suggested wide range of serum iPTH levels (2–9 fold) in the guideline is difficult to apply for health providers in their clinical settings [15,16].

To better quantify the optimal levels of serum iPTH for a lower risk of mortality, we examined the association between different serum iPTH levels and mortality among a group of incident HD patients. We hypothesized that there would be a different risk of morality among HD patients with different levels of serum iPTH.

## Materials and methods

### Study design and population

The study protocol was approved by the Institutional Ethical Review Board of Beijing Shijitan Hospital, Capital Medical University (SJT2020-18), and the procedures were conducted in following the Good Clinical Practice and the Declaration of Helsinki on biomedical research involving human subjects. All participants provided informed consent.

The study participants were hemodialysis patients recruited from the dialysis center of Beijing Shijitan Hospital, Capital Medical University between April 2010 and June 2015. The inclusion criteria were as follows: (1) age ≥18 years, (2) newly diagnosed with end-stage renal disease (ESRD) and starting hemodialysis for 3 months. The exclusion criteria were: (1) unwilling to provide written informed consent, (2) inadequate baseline data available for analysis.

### Data collection and laboratory measurements

After enrollment, we collected the participants’ demographic data, smoking habits, body mass index (BMI), the etiology of ESRD, comorbidities, medications with outcome influences, and type of vascular access. The blood tests included measurement of the serum levels of hemoglobin, albumin, triglycerides, total cholesterol, high sensitivity C-reactive protein (hs-CRP), calcium, phosphate, iPTH, and 25(OH)VitD. Blood samples were collected on the day of the first dialysis session in a week. Serum iPTH was measured by an electro chemiluminescence immunoassay run on a DXI800 auto analyzer (Beckman Coulter, CA, USA; reference range 15–75 pg/mL). Urea clearance index single-pool *K_t_*/V (sp *K_t_*/V) was calculated by the pre- and post-dialysis serum urea nitrogen levels. The estimated glomerular filtration rate (eGFR) was calculated by a modified Chronic Kidney Disease Epidemiology Collaboration (CKD-EPI) formula with an adjusted coefficient of 1.1 for the Chinese population [17].

### Outcome measurements

The primary outcomes were all-cause mortality and cardiovascular disease (CVD) mortality. The CVD mortality was defined as death from CVD including sudden cardiac death, myocardial infarction, congestive heart failure, and stroke. Survival time was defined as the time elapsed from initial study enrollment until death, modality switch to peritoneal dialysis, kidney transplantation, or the end of the study period (31 January 2020).

### Statistical methods

We used IBM SPSS Statistics 21.0 (IBM Corp., Armonk, NY) to perform the analyses. Descriptive analysis, including proportions, means, and frequencies, was used to define participant characteristics. Continuous variables were expressed as mean with standard deviation (SD) or median with interquartile range (IQR); categorical variables were expressed as a number with a percentage. Comparisons between groups were performed with one-way ANOVA for normally distributed continuous variables or Kruskal-Wallis H tests for skew distributed continuous variables, and Chi-square test for categorical data.

We generated univariate and multivariable Cox proportional hazards regression analysis to determine the crude and adjusted hazard ratios (HR) for all-cause mortality and CVD mortality. The multivariable Cox analysis was adjusted for demographic characteristics (age, gender), BMI, comorbidities (diabetes, hypertension), MBD-related medications (phosphate binders, vitamin D, and Cinacalcet), type of vascular access, laboratory parameters (hemoglobin, albumin, triglycerides, total cholesterol, hs-CRP, calcium, phosphate, iPTH, 25(OH)VitD), eGFR, and dialysis parameters (sp *K_t_*/V). In the adjustment analysis, serum iPTH, calcium, and phosphate were divided into clinically relevant categories according to the K/DOQI [10] target ranges. Serum iPTH 150–300 pg/mL, serum calcium 2.10–2.37 mmol/L, and phosphate 1.13–1.78 mmol/L were used as a reference, respectively.

We also performed a sensitivity analysis of patients divided into six groups of different serum iPTH levels according to the folds of the K/DOQI target range of serum iPTH, i.e. serum iPTH < 150 pg/mL, 150–299 pg/mL, 300–449 pg/mL, 450–599 pg/mL, 600–749 pg/mL, and ≥750 pg/mL, respectively. We repeated the multivariable Cox regression analysis by using the group with serum iPTH 150–299 pg/mL as the reference group. A *p* < 0.05 was considered statistically significant.

## Results

### Baseline demographic and clinical characteristics of the study population

The total study sample surveyed included 362 incident HD patients. After excluding 10 patients with inadequate clinical or laboratory data and 6 patients who were not willing to participate, 346 hemodialysis patients were included in the final analysis. The overall enrollment rate was 95.58%. The mean age of participants was 62.08 ± 14.56 years (30–85 years), 182 patients (52.60%) were men. The primary etiology of ESRD was diabetic nephropathy (*n* = 128, 36.99%), followed by chronic glomerulonephritis (*n* = 125, 36.13%), hypertensive renal disease (*n* = 37, 10.69%), chronic tubulointerstitial nephropathy (*n* = 33, 9.54%), and others (*n* = 23, 6.65%). The dialyzers with polyacrylonitrile, polysulfone, polycarbonate membranes used in the hemodialysis treatment were 75.50%, 13.80%, and 10.70%, respectively. The average treatment session length was 3.85 ± 0.22 h. Calcium dialysate concentration was 1.5 mmol/L. The mean iPTH levels were 332.40 ± 296.56 pg/mL (10–1921 pg/mL). 110 patients (31.79%) were categorized as iPTH < 150 pg/mL group, 94 patients (27.17%) as 150 ≤ iPTH ≤ 300 pg/mL group, and 142 patients (41.04%) as iPTH > 300 pg/mL group. The baseline characteristics were shown in Table 1. The age, BMI, the proportion of patients with diabetes mellitus, proportion of used phosphate binders, blood hemoglobin, serum albumin, triglycerides, total cholesterol, hs-CRP, phosphorus, and *K_t_*/V differed among the three groups. In contrast, there were no significant differences among the groups in terms of gender, smoking habit, prevalence of hypertension, the type of vascular access, serum calcium, 25(OH)VitD, and eGFR.

**Table 1.** Baseline characteristics of study participants.

Characteristics	Total (*n* = 346)	iPTH < 150 pg/mL (*n* = 110)	150≤iPTH≤300 pg/mL (*n* = 94)	iPTH > 300 pg/mL (*n* = 142)	*p*-Value
Age, (years)	62.08 ± 14.56	66.02 ± 9.99	61.68 ± 13.93	59.25 ± 14.71	<0.001
Male sex, *n* (%)	182 (52.60)	56 (50.91)	58 (61.70)	68 (47.89)	0.105
Smoking, *n* (%)	106 (30.64)	29 (26.36)	32 (34.04)	45 (31.69)	0.465
BMI (kg/m^2^)	22.67 ± 3.42	22.28 ± 3.63	22.09 ± 3.44	23.34 ± 3.13	0.008
Diabetes mellitus, *n* (%)	194 (56.07)	86 (78.18)	46 (48.94)	62 (43.66)	<0.001
Hypertension, *n* (%)	294 (84.97)	92 (83.64)	74 (78.72)	128 (90.14)	0.050
Medication use					
Phosphate binders, *n* (%)	204 (58.96)	52 (47.27)	48 (51.06)	104 (73.24)	<0.001
Vitamin D, *n* (%)	146 (42.20)	50 (45.46)	36 (38.30)	60 (42.25)	0.587
Cinacalcet, *n* (%)	37 (10.69)	11 (10.00)	10 (10.64)	16 (11.27)	0.949
Vascular access type					
Arteriovenous fistula, *n* (%)	170 (49.13)	50 (45.46)	51 (54.26)	69 (48.59)	0.450
Catheter, *n* (%)	174 (50.30)	59 (53.64)	42 (44.68)	73 (51.41)	0.418
Other, *n* (%)	2 (0.58)	1 (0.91)	1 (1.06)	0	0.491
Laboratory parameters					
Hemoglobin (g/L)	110.92 ± 16.20	107.35 ± 15.91	111.43 ± 15.61	113.37 ± 16.42	0.013
Albumin (g/L)	37.82 ± 4.50	34.99 ± 4.14	38.09 ± 4.94	39.83 ± 3.14	<0.001
Triglycerides (mmol/L)	2.08 ± 1.14	1.97 ± 0.89	1.87 ± 1.05	2.30 ± 1.31	0.009
Total cholesterol (mmol/L)	4.33 ± 1.04	4.55 ± 1.00	4.20 ± 1.25	4.25 ± 0.90	0.028
hs-CRP (mg/L)	7.93±5.53	10.18 ± 7.46	7.02 ± 4.45	6.79 ± 3.59	<0.001
Calcium (mmol/L)	2.23 ± 0.20	2.25 ± 0.17	2.22 ± 0.20	2.23 ± 0.21	0.405
Phosphorus (mmol/L )	1.98 ± 0.63	1.54 ± 0.35	1.84 ± 0.61	2.40 ± 0.55	<0.001
iPTH (pg/ mL)	259 (118.448)	81 (51.116)	220 (173.280)	541 (393.646)	<0.001
25 (OH)VitD (mg/mL)	9.25 ± 3.71	9.66 ± 3.45	8.60 ± 3.39	9.37 ± 4.07	0.114
eGFR (ml/min/1.73 m^2^)	6.724 ± 0.587	6.646 ± 0.611	6.756 ± 0.509	6.724 ± 0.587	0.321
sp *K_t_*/V	1.39 ± 0.25	1.34 ± 0.21	1.40 ± 0.24	1.42 ± 0.27	0.036

Data were presented as mean ± SD or median (interquartile range) for continuous variables, and number (%) for categorical variables. BMI: body mass index; hs-CRP: high sensitive C-reactive protein; iPTH: intact parathyroid hormone; 25 (OH)VitD: 25-hydroxy vitamin D.

### Association of serum iPTH levels and mortality

The median follow-up was 58 months. There were 157 (45.38%) cases of death during the follow-up. The primary causes of death were CVD (109, 69.43%), followed by infection (25, 15.92%), and other diseases caused by malignancy, chronic obstructive lung disease, gastrointestinal bleeding, and others (23, 14.65%). Table 2 presented the risk factors associated with all-cause mortality and CVD mortality (Supplementary Table). By using the group with serum iPTH 150–300 pg/mL as the reference group, multivariate adjusted Cox regression analysis showed that either iPTH < 150pg/mL or iPTH > 300 pg/mL were independently associated with higher hazard ratio for all-cause mortality (HR 4.022, 95% CI 2.493–6.489, *p* < 0.001 and HR 1.752, 95% CI 1.008–3.044, *p* = 0.047, respectively) and CVD mortality (HR 4.729, 95% CI 2.535–8.822, *p* < 0.001 and HR 1.867, 95% CI 1.007–3.565, *p* = 0.049, respectively).

**Table 2.** Multivariable Cox regression analyses for the risk factors of mortality.

Characteristics	All-cause mortality		CVD mortality	
	HR (95%CI)	*p*-Value	HR (95%CI)	*p*-Value
Age, (years)	1.025 (1.010–1.039)	0.001	1.032 (1.015–1.050)	<0.001
History of				
Diabetes	1.658 (1.115–2.466)	0.013	2.934 (1.688–5.100)	<0.001
Hypertension	–	–	2.540 (1.315–4.908)	0.006
Vitamin D used	0.573 (0.370–0.888)	0.013	–	–
sp *K_t_*/V	0.089 (0.038–0.208)	<0.001	0.048 (0.018–0.129)	<0.001
Hemoglobin (g/L)	0.980 (0.969–0.991)	<0.001	0.975 (0.961–0.988)	<0.001
Albumin (g/L)	0.923 (0.883–0.965)	<0.001	–	–
Calcium				
<2.10 mmol/L	1.699 (1.162–2.485)	0.006	–	–
2.10–2.37 mmol/L	1.000 (reference)		–	–
>2.37 mmol/L	0.920 (0.602–1.405)	0.698	–	–
Phosphorus				
<1.13 mmol/L	0.702 (0.454–1.087)	0.112	0.957 (0.580–1.484)	0.092
1.13–1.78 mmol/L	1.000 (reference)		1.000 (reference)	
>1.78 mmol/L	1.674 (1.117–2.507)	0.013	1.560 (1.098–2.484)	0.014
iPTH				
<150 pg/mL	4.022 (2.493–6.489)	<0.001	4.729 (2.535–8.822)	<0.001
150–300 pg/mL	1.000 (reference)		1.000 (reference)	
>300 pg/mL	1.752 (1.008–3.044)	0.047	1.867 (1.007–3.565)	0.049

Model was adjusted for age, gender, BMI, comorbidities of diabetes and hypertension, medications (phosphate binders, vitamin D, and Cinacalcet), vascular access type, the serum level of hemoglobin, albumin, triglycerides, total cholesterol, hs-CRP, calcium, phosphate, iPTH, 25 (OH)VitD, eGFR, and sp *K_t_*/V. CVD: cardiovascular disease; HR: hazard ratio; CI: confidence interval; iPTH: intact parathyroid hormone.

### The optimal serum iPTH range associated with the lowest risk of mortality

**Figure 1.**
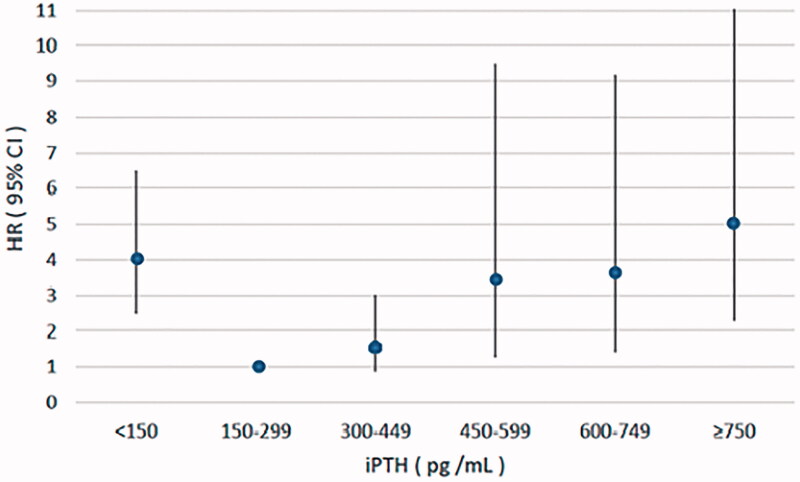
Multivariate adjusted hazard ratio (95% CI) for all-cause mortality according to the levels of serum iPTH. iPTH: intact parathyroid hormone; HR: hazard ratio; CI: confidence interval.

**Figure 2.**
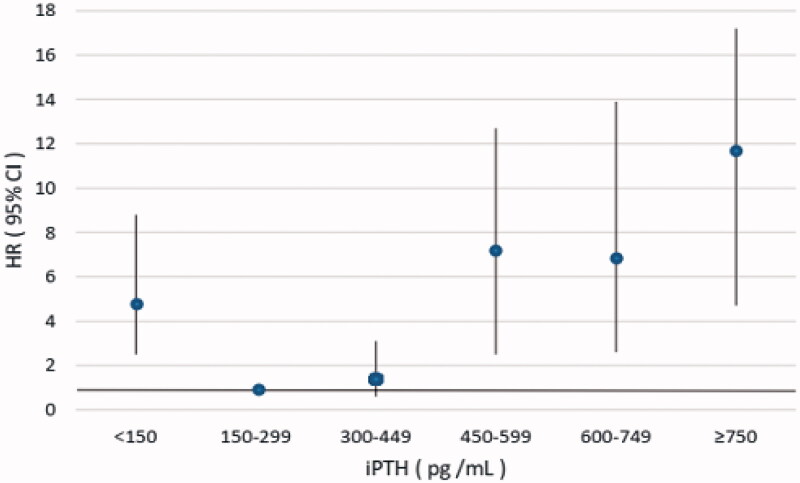
Multivariate adjusted hazard ratio (95% CI) for CVD mortality according to the levels of serum iPTH. iPTH: intact parathyroid hormone; HR: hazard ratio; CI: confidence interval.

In the sensitivity analysis, we further divided the patients into six serum iPTH groups. Multivariate adjusted Cox analysis result showed that patients with serum iPTH ≥750 pg/mL, 600–749 pg/mL, 450–599 pg/mL had significantly higher risk of all-cause mortality and cardiovascular mortality compared with those with serum iPTH in the range of 150–299pg/mL, while patients with iPTH 300–449 pg/ml had no significant difference (Table 3, Figures 1 and 2).

**Table 3.** Multivariable Cox regression analyses for mortality according to the different range of serum iPTH.

iPTH (pg/mL)	All-cause mortality			CVD mortality		
	No. (%) of event	HR (95%CI)	*p*-Value	No. (%) of event	HR (95%CI)	*p*-Value
<150	82 (74.55)	4.022 (2.493–6.489)	<0.001	56 (50.91)	4.729 (2.535–8.822)	<0.001
150–299	28 (29.79)	reference 1.000		15 (15.96)	reference 1.000	
300–449	20 (33.33)	1.614 (0.861–3.027)	0.135	11 (18.33)	1.373 (0.603–3.127)	0.450
450–599	7 (23.33)	3.475 (1.270–9.505)	0.015	7 (23.33)	7.16 (2.475–12.735)	<0.001
600–749	8 (30.77)	3.605 (1.416–9.177)	0.007	8 (30.77)	6.817 (2.594–13.912)	<0.001
≥750	12 (46.15)	5.016 (2.278–11.043)	<0.001	12 (46.15)	11.745 (4.726–17.187)	<0.001

Model was adjusted for age, gender, BMI, comorbidities of diabetes and hypertension, medications (phosphate binders, vitamin D, and Cinacalcet), vascular access type, the serum level of hemoglobin, albumin, triglycerides, total cholesterol, hs-CRP, calcium, phosphate, iPTH, 25 (OH)VitD, eGFR, and sp *K_t_*/V. CVD: cardiovascular disease; HR: hazard ratio; CI: confidence interval; iPTH: intact parathyroid hormone.

## Discussion

In this prospective observational study, we found that the lower level of serum calcium, a higher level of serum phosphorus, and a lower or higher level of serum iPTH of the incident HD patients were associated with all-cause mortality and CVD mortality even after adjustment of confounding factors. These findings suggested a *U*-shaped association between serum iPTH and mortality. Furthermore, we demonstrated that the optimal serum iPTH for a relatively lower risk of all-cause mortality and CVD mortality were from 150 pg/mL to 450 pg/mL in those patients. Our result provided evidence for the proper range of serum iPTH among the incident HD patients.

Previous observational data suggested an association between abnormal biochemical markers of mineral metabolism and death [18–22]. However, the results of these studies were inconsistent [23–28]. Covic et al. [29] made a meta-analysis with 19 studies that assessed the association between disturbances in biochemical parameters and all-cause mortality among patients on dialysis. They found that elevated values in certain biochemical parameters, specifically the serum levels of iPTH, calcium, and phosphorus, as well as very low values of phosphorus were associated with an increase in all-cause mortality. In contrast to Covic et al., Palmer et al. [30] concluded from their meta-analysis that calcium and PTH did not have statistically significant associations with mortality, and only elevated serum phosphorus was related to mortality. The authors noticed that the studies were too methodologically diverse to permit an appropriate meta-analysis. Most importantly, both of these studies assumed a linear relationship between the serum levels of the three biochemical parameters and all-cause mortality in their meta-analysis, which could have biased the results of them. To avoid this limitation, we divided those three CKD-MBD biochemical parameters into three groups according to the recommendation from the K/DOQI guideline [10] and used them as categorical variables in the Cox regression analyses in our study. Our results indicated that elevated levels of serum phosphorus, decreased calcium, and either elevated or decreased serum iPTH were associated with death among these HD patients. Our study, in accordance with some previous studies [5,29,31] suggested that the relationship between the serum iPTH levels and mortality appeared as *U*-shaped, which indicated that the serum iPTH of the HD patients should keep in a certain range so that they could have a relatively lower risk of death.

Although the KDIGO guideline for CKD-MBD has suggested maintaining serum iPTH level in approximately two to nine times the upper normal limit, such a wide range of serum iPTH suggestion may bring confusion in clinical practice and the recommendation level was only C, which calls for related supporting evidence on this issue. To clarify this uncertainty, we made a sensitivity analysis to determine the optimal serum iPTH levels for a lower risk of all-cause mortality and CVD mortality in our patients. Our findings showed a lower risk of death in patients with iPTH in the range of 150–450 pg/mL. This serum iPTH range was narrower than that of the KDIGO guideline. When the serum iPTH was greater than 450 pg/mL, the mortality increased significantly; similarly, the mortality was 4 folds in the group of patients with serum iPTH < 150 pg/mL compared with those whose serum iPTH was 150–299 pg/mL. Recently, Hong et al. [9] showed that patients with iPTH < 150 pg/mL (*n* = 582) had a higher risk of infection-related mortality in 1771 incident dialysis patients. Furthermore, Merle and colleagues showed that patients with iPTH levels < 2 times the upper limit of normal values for the measurement kit used induced by high dialysate calcium is an independent risk factor for CVD mortality in hemodialysis patients [25]. These data not only partially supported our results about the U-shaped relationship between serum iPTH and mortality but also reminded us that we should pay more attention to prevent adverse outcomes among patients who have lower levels of serum iPTH.

There are several limitations to this study. First, as a prospective single-center study, it might have introduced selection bias, and the results should be carefully extrapolated to other HD patients. However, single-center study has some advantages of the similarity of the quality control in the whole process of the study. Second, we did not assess other biochemical markers related to CKD-MBD, such as fibroblast growth factor-23, which might cause interaction with the current biomarkers like serum calcium and phosphorus, and these data should be added in the future study. Thirdly, we only used the entry data to assess the association of CKD-MBD-related biomarkers (serum calcium, phosphate, and iPTH) with mortality, perhaps the time-average values of these parameters would give us a more meaningful hint about the relationship between CKD-MBD and adverse outcomes. Finally, causality cannot be inferred in this observational study. Interventional studies are needed to further clarify whether the restriction of serum iPTH at a certain optimal level could be a benefit to reduce the mortality among those patients. Regardless of these study limitations, several strengths of the present study are worth highlighting. Our observations are of importance as this is a prospective observational study about the relationship between serum iPTH level and all-cause mortality and CVD mortality in Chinese patients undergoing hemodialysis. The results were based on observation over a long period of follow-up (median follow-up of 58 months). To the best of our knowledge, it is conducted to date with the longest follow-up period examining the optimal serum iPTH level with improved outcomes among Chinese incident HD patients.

## Conclusion

In the current study, we conclude that the relationship between serum iPTH and mortality appears to be a U-shaped curve. The optimal serum iPTH level which confers the lowest risk of all-cause mortality and CVD mortality ranges from 150 pg/mL to 450 pg/mL in this group of Chinese incident HD patients. We should pay more attention to HD patients whose serum iPTH level are extremely low or high, even in the range of serum iPTH level provided by the KIDGO guideline.

## Supplementary Material

Supplemental Material
