# Supplementary material for: The optimal range of serum intact parathyroid hormone for a lower risk of mortality in the incident hemodialysis patients
Source: Ren Fail. 2021 Mar 29;43(1):599–605. doi: 10.1080/0886022X.2021.1903927 (PMC8018348; doi:10.1080/0886022X.2021.1903927)
Supplement: Supplemental Material [file IRNF_A_1903927_SM3442.pdf]

Supplementary table. Multivariable Cox regression analyses for all-cause mortality and CVD mortality according to the different level of serum phosphorus in patients with iPTH150-450pg/mL.

**Phosphorus (mmol/L)**	**All-cause mortality**			**CVD mortality**		
	No. (%) of event	HR (95%CI)	*p*-value	No. (%) of event	HR (95%CI)	*p*-value
**<1.13**	12(41.38)	2.103 (0.944-4.683)	0.069	8(27.59)	2.200 (0.641-7.547)	0.210
**1.13-1.78**	12(20.69)	reference		6(10.34)	reference	
**>1.78**	24(35.82)	2.022(1.010-4.047)	0.047	12(17.91)	5.192 (1.590-16.946)	0.006

Model was adjusted for age, gender, BMI, comorbidities of diabetes and hypertension, medications (phosphate binders, vitamin D, and Cinacalcet), vascular access type, the serum level of hemoglobin, albumin, triglycerides, total cholesterol, hs-CRP, calcium, phosphate, iPTH, 25(OH)VitD, eGFR, and sp Kt/V. CVD: cardiovascular disease; HR: hazard ratio; CI: confidence interval; iPTH: intact parathyroid hormone.
